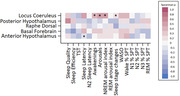# Subcortical correlates of sleep across Alzheimer’s Disease and cognitively healthy adults: an in vivo study

**DOI:** 10.1002/alz.085382

**Published:** 2025-01-03

**Authors:** Neus Falgàs Martínez, Andrea Val‐Guardiola, Gerard Mayà, Marta Peña, Guadalupe Fernandez‐Villullas, Beatriz Bosch‐Capdevila, Albert Lladó, Emma Muñoz‐Moreno, Álex Iranzo, Lea T. Grinberg, Raquel Sanchez‐Valle

**Affiliations:** ^1^ Alzheimer’s disease and other cognitive disorders unit, Hospital Clínic, IDIBAPS, Barcelona Spain; ^2^ Hospital Clínic, Barcelona Spain; ^3^ IDIBAPS, Barcelona, Barcelona Spain; ^4^ Alzheimer’s Disease and Other Cognitive Disorders Unit, Hospital Clínic, Institut d’Investigacions Biomediques August Pi i Sunyer (IDIBAPS), Barcelona Spain; ^5^ Image Diagnostic Centre, IDIBAPS, Hospital Clínic de Barcelona, Barcelona, Spain, Barcelona Spain; ^6^ Neurology Service, Hospital Clínic de Barcelona and Institut D'Investigacions Biomèdiques August Pi i Sunyer (IDIBAPS), Barcelona Spain; ^7^ Weill Institute for Neurosciences, University of California San Francisco, San Francisco, CA USA; ^8^ Alzheimer’s disease and other cognitive disorders Unit. Hospital Clínic de Barcelona; FRCB‐IDIBAPS; University of Barcelona, Barcelona Spain

## Abstract

**Background:**

Neuromodulatory subcortical systems (NSS) are affected from the early stages of Alzheimer’s Disease (AD) by the accumulation of tau pathology. Increased tau burden within the subcortical nucleus that are in control of sleep and wake regulation may contribute to the breakdown of sleep‐wake patterns in AD. A recent postmortem study showed that subcortical wake‐promoting neurons were related to sleep phenotypes in AD and PSP, being that greater neuronal count in locus coeruleus (LC), tuberomammillary nucleus (TMN), and lateral hypothalamic area (LHA) associated with a decreased sleep drive (Oh et al., 2022). However, evidence studying this relationship in *vivo* is still lacking. The current study examines the association between sleep metrics and wake/sleep‐promoting nuclei using polysomnography and MRI.

**Methods:**

Forty‐three subjects with AD biomarker‐based diagnosis and 15 cognitively healthy individuals with normal pTau plasma levels were recruited at the Hospital Clínic de Barcelona. All participants completed the Epworth Sleepiness Scale (ESS) and Pittsburg Sleep Quality Index (PSQI). All participants underwent overnight polysomnography and a 3T MRI scan, including T1 and *fast spin‐echo* sequences. We measured volumes of wake‐promoting nuclei: LC, posterior hypothalamus (including TMN and LHA), dorsal raphe and basal forebrain, and sleep‐promoting nucleus: anterior hypothalamus (including preoptic area and paraventricular nucleus) (Billot et al., 2020; Rolls et al., 2020; Greve et al., 2020). Correlation analysis between sleep metrics and subcortical volumes were performed.

**Results:**

Preliminary results from 25 AD participants and 2 healthy controls showed that wake‐promoting nuclei volumes were predominantly associated with measures indicating decreased sleep drive (*Figure 1*): Greater LC volumes associated with increased awakenings (*p*<0.01), arousals (*p*<0.05), number of sleep stage changes (*p*<0.05) and NREM arousal index (*p*<0.05). Regarding the sleep‐promoting nucleus, greater volumes of the anterior hypothalamus were associated with decreased sleep latency (*p*<0.05). No significant correlations were found with sleep questionnaires. Further analyses increasing the sample size will be performed.

**Conclusions:**

The current preliminary study suggests that the subcortical system is a primary mechanism associated with sleep disturbances in the early stages of neurodegenerative diseases, reinforcing previous neuropathological findings.